# Empirical Performance and Energy Consumption Evaluation of Container Solutions on Resource Constrained IoT Gateways

**DOI:** 10.3390/s21041378

**Published:** 2021-02-16

**Authors:** Syed M. Raza, Jaeyeop Jeong, Moonseong Kim, Byungseok Kang, Hyunseung Choo

**Affiliations:** 1 Department of Electrical and Computer Engineering, Sungkyunkwan University, Suwon 16419, Korea; 2 Department of Computer Science and Engineering, Sungkyunkwan University, Suwon 16419, Korea; jaeyeop@skku.edu; 3 Department of Liberal Arts, Seoul Theological University, Bucheon 14754, Korea; moonseong@stu.ac.kr; 4 Department of Electronics, Computing and Mathematics, University of Derby, Derby DE22 1FX, UK; b.kang@derby.ac.uk

**Keywords:** containers, docker swarm, kubernetes, energy consumption, empirical analysis, Internet of Things

## Abstract

Containers virtually package a piece of software and share the host Operating System (OS) upon deployment. This makes them notably light weight and suitable for dynamic service deployment at the network edge and Internet of Things (IoT) devices for reduced latency and energy consumption. Data collection, computation, and now intelligence is included in variety of IoT devices which have very tight latency and energy consumption conditions. Recent studies satisfy latency condition through containerized services deployment on IoT devices and gateways. They fail to account for the limited energy and computing resources of these devices which limit the scalability and concurrent services deployment. This paper aims to establish guidelines and identify critical factors for containerized services deployment on resource constrained IoT devices. For this purpose, two container orchestration tools (i.e., Docker Swarm and Kubernetes) are tested and compared on a baseline IoT gateways testbed. Experiments use Deep Learning driven data analytics and Intrusion Detection System services, and evaluate the time it takes to prepare and deploy a container (creation time), Central Processing Unit (CPU) utilization for concurrent containers deployment, memory usage under different traffic loads, and energy consumption. The results indicate that container creation time and memory usage are decisive factors for containerized micro service architecture.

## 1. Introduction

Internet of Things (IoT) devices are becoming ubiquitous in our daily lives. Typically, IoT devices consist of sensors which collect data and send it to the cloud for further processing and analysis [1,2]. In last few years, IoT based realtime consumer services have rapidly increased their market share, and they have tight latency requirements to maintain users’ quality of experience. Most representative example services with tight latency requirements are virtual home assistants like Amazon Echo, and security devices such as smart doorbell and cameras [3]. A major issue in these service scenarios is to promptly process the data in IoT devices under given latency constraints with low energy consumption. Mostly, computation and power resources in IoT devices are severely constrained and cannot meet the given latency requirements. In literature this problem is tackled by offloading the computation to fog devices which are deployed at the network edge under fog computing architecture [4,5,6].

Fog computing is a horizontal architecture in which computing, storage, networking functions, and control are distributed closer to the users (i.e., network edge) in the form of fog nodes [7]. A fog node consists of computing servers, storage devices, and a network to connect them all. IoT gateway is an essential part of each fog node [8] as it provides connectivity to IoT devices and performs several other critical functions like protocol translation, encryption, data filtering, and lastly data computing. As IoT gateway is a closest computing resource available to IoT devices for offloading their tasks, it can provide several micro-services and orchestrate intelligent applications and services by using different Artificial Intelligence (AI) algorithms. For example, deployment of AI based data analytics services like motion tracking, speech synthesis, and location prediction on IoT gateway can improve IoT system performance. Similarly, firewall and Intrusion Detection System (IDS) on IoT gateways can detect and protect IoT systems against cyber-attacks with great effect [9,10,11]. However, IoT gateways are also resource constrained and require these services to be dynamically deployed, scaled, and removed through the use of virtualization technologies like containers [12,13].

Container is a software package that bundles applications’ code with its runtime environment that includes libraries, configurations, and dependencies to start-up, migrate, and shutdown application more quickly [14]. A container uses operation system level virtualization to use host resources in isolation from other containers, thus, it has much less overhead comparing to Virtual Machines (VMs) [15,16]. There are several container solutions such as Dockers, Linux Containers, Solaris Zone, and RKT, which are orchestrated through tools like Kubernates (K8s), Docker Swarm (DS), and Amazon ECS. Performance of containers and their orchestrators is critically important for resource constrained environments. Recent studies have analyzed containers in resource constrained devices from different aspects, such as, deployment architecture [17], computation performance [15,18], orchestration [19], service scalability [20,21], and energy consumption [22,23]. Experiments in these studies are done for resourceful devices under moderate conditions, and are not applicable to resource constrained IoT gateways/devices. Moreover, these studies are limited to a particular aspect/application of containers, where neither they have analyzed all facets of containers performance nor provided comparison between different container technologies and orchestration tools.

This paper distinguishes itself from the previous studies through evaluation and comparison of two container orchestration tools for allocation and management of containers in resource constrained IoT gateways. DS and K8s orchestration tools are chosen for the evaluation, where the former is pioneering orchestration platform and the later is a current industry leader, as per market survey of more than 400 IT professionals reported in [24]. Service deployment in the experiments uses these orchestration tools for the deployment of respective containers on IoT gateways made of Raspberry Pi boards. Multiple open source data analytic services using Deep Learning (DL) and IDS service containers are used in the experiments to determine the container creation time, increment rate in CPU utilization for concurrent container deployments, memory usage under different traffic loads, and energy consumption. Zabbix, an open source monitoring system, is utilized to gather and analyze measurements from these experiments [25]. The contribution of this paper is to design a containerized services deployment guideline for operators through comprehensive experiments. This allows operators to design more efficient and cost-effective dynamic service provisioning systems. Additionally, presented results are not only applicable for IoT gateways but are also relevant for IoT devices, as the experimentation platform is based on Raspberry Pi. Hence, the results also include energy consumption comparison due to its critical importance in IoT devices.

The remaining paper is organized as follows. Section 2 describes the background of DS and K8s, and current studies related to performance of containers and service architectures for IoT devices. Section 3 explains the testbed architecture, containers deployment environment, and different experiment scenarios. Section 4 presents the discussion on detailed evaluation and comparison of DS and K8s. Lastly, Section 5 presents the conclusion and outlines the key factors for successful and cost-effective containerized service provisioning in resource constrained IoT devices.

## 2. Related Studies and Background Discussion

The first part of the section reviews current studies that evaluate different performance aspects of container orchestration tools. The later part presents the architectures and essential functions of DS and K8. There are various open source container orchestration solutions available, and different market survey reports show different orchestration solutions as market leader. However, DS and K8s are among the top container orchestration tools in all of the reports that authors have surveyed [24,26,27].

### 2.1. Literature Review

A container orchestration tool automates the deployment, scaling, management, and networking of containers. In the last few years several container orchestration tools are launched, such as DS, K8s, Amazon ECS, Distributed Cloud Operating System (DC/OS) based on Apache Mesos, and Azure Service Fabric. All these orchestration tools have their advantages and disadvantages as they emphasize one aspect of container management over the others. A recent study compares DS, K8s, and DC/OS in terms of supported functionalities, genericity, maturity, and stability [28]. It divides functionalities into nine functional aspects and 27 sub-aspects, and K8s shows highest number of common and unique features in all nine functional aspects but all three tools are similar in terms of genericity. This qualitative comparison helps in selecting an orchestration tool based on features but lacks performance insight, and it is not feasible to select an orchestration tool based on only features and maturity. To make a performance oriented selection, a study compares services provisioning time in DS, K8s, Apache Mesos, and Cattle [29]. The experiments are done with three applications of increasing complexity and provisioning time is recorded for local images and remote images in Docker registry. The results show that K8s has best performance in case of local images of application but it drops in case of remote images, and this is because of communication overhead between K8s agent and Docker registry.

A more in depth performance comparison between DS and K8s is done in [30]. This study separately deploys a web based micro-service named MARKA [31] on a server machine using DS and K8s, and measures the response time as the number of users are increased from 100 to 500. The response time is individually measured for each of the 10 operations in MARKA application, and results show that for 100 to 200 users DS response time is significantly less than K8s for almost every operation. However, as the number of users increases to 500, the response time gap between DS and K8s shrinks, but DS still has overall less response time than K8s has. A similar study targeting fog computing in IoT systems context is presented in [32]. Authors in this study have used different Small Board Computers (SBCs) such as Raspberry Pi 3, Udoo, Tinker, and Jetson to make a fog cluster where one device is master and others are slave. The master device divides audio analysis tasks to other slave devices, where DS and K8s based containers analyze audio chunks for psycho-acoustic parameters such as loudness, sharpness, roughness, and fluctuation strength to other slave devices. During the analysis CPU and RAM usages and CPU temperature are measured, and experimental results show that DS outperforms K8s in both resource usage and processing time. Although this study provides comprehensive comparison between DS and K8s, no clear deployment guidelines are provided for the operators to help them in designing their micro-service architectures.

Energy consumption is an important factor for resource constrained IoT devices, and must be taken into account for containerized services deployment.A comprehensive study compares different hardware platforms for IoT devices by using native and docker container applications [22]. The study includes power and energy consumption evaluations by using different benchmark tools, and concludes that Odroid C2 outperforms other single board computer devices in most of the experiments. Another study uses a real application like OpenCV with different configurations to determine the performance and energy consumption of Raspberry Pi [33]. The results show the execution time and energy consumption when CPU frequency is increased from 400 MHz to 1.2 GHz, and for different configurations results vary significantly and no clear pattern emerges. Therefore, no conclusive arguments are made in this study regarding execution time and energy consumption. In addition to experimentation based evaluations, power consumption models are created for various components of Raspberry Pi through experiment results under different load conditions [23]. The presented power consumption models include models for individual components like CPU, memory, and network, and a model for overall power consumption of Raspberry Pi.

In summary, the studies related to comparison of containers orchestration tools are mostly done for resourceful cloud platforms and do not consider resource constraint aspects of IoT devices. The comparative studies, which are particularly done for IoT devices, lack final discussion to point out which orchestration tool is beneficial in a particular deployment scenario. Our previous study [34] outlines the performance of containerized services using DS in IoT gateways testbed based on Raspberry Pi boards. This paper extends the testbed to perform a comparative analysis between DS and K8s for creation time of containers, CPU utilization of concurrent container deployments, memory usage, and energy consumption. The experiments and their analysis are done with an intention to provide base guidelines for micro-services deployment, which can facilitate operators to efficiently design and manage their networks for delay sensitive services.

### 2.2. Docker Swarm (DS) and Kubernetes (K8s)

**Figure 1:**
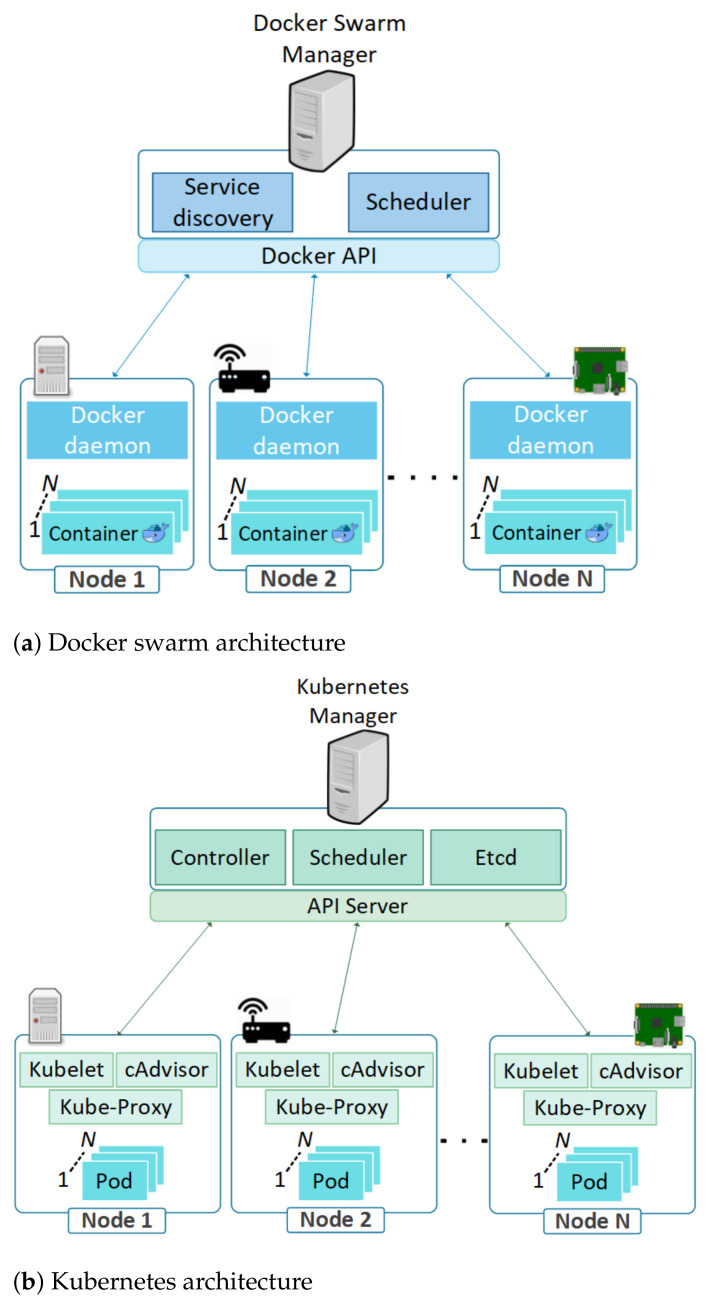
Docker swarm architecture

In a large-scale container deployment over numerous servers, keeping track of all the containers and managing their life cycles are essential and complex tasks. DS is an open source orchestration platform for administering services life-cycles in massive deployments of docker containers. Compatibility of DS with existing Docker command line interface and configuration tools facilitates a smooth progression from native Docker to DS. In comparison to Network Function Virtualization (NFV) management frameworks, DS is architecturally simple and comprises of a manager which controls numerous nodes, as shown in Figure 1a. Two notable modules in the manager are scheduler and service discovery. Service discovery module maintains the details about the nodes with containers that are operational. Monitoring of available resources in nodes with operational containers, and allocation of newly created containers in the most appropriate node are done by the scheduler modules. The containers management operations in a node are taken care of by a daemon process. It functions as part of host OS and is responsible for overall container state management, for instance IP connectivity, connections, and functional status [35].

Similar to DS, K8s is a container cluster orchestration tool created by Google. It is optimized for managing massive servers and container deployments that are larger than Docker Swarm. K8s architecture in Figure 1b shows a master which manages numerous nodes. Nodes consist of clusters of K8s and master manages them through a controller manager, scheduler, Etcd, and API server. Controller manager manages the cluster using API as a tool that combines various managers into a single file. Scheduler distributes containers to nodes and Etcd stores information about states of the entire cluster. K8s architecture consists of pods where each pod has one or several containers inside it [36]. A node is a server running pod and consists of Kubelet, CAdvisor, and Kube-proxy. CAdvisor monitors containers while Kubelet communicates with the API server for overseeing operations of running pods [37]. Kube-proxy is responsible for the network communications of the nodes, and helps the pods to communicate within the node.

## 3. System Design

### 3.1. IoT Services

At the network edge, each IoT gateway is the first point of the data collection pipeline which ends at the cloud. Prompt computation of the collected data is essential for delay sensitive services which are becoming common in home automation, autonomous personal assistants, and industry automation. Data analysis requires statistical algorithms, and recently learning based algorithms have also been used for this purpose. It is essential to determine the performance of these algorithms in containers under limited resources of IoT gateways. Tensorflow [38], Keras [39], and Pytorch [40] are open source learning frameworks with high usage rates. Tensorflow is a Python based deep learning framework developed by Google that can function on all OSes. Keras is also a Python based, and it functions as an API over Tensorflow with a quick provision of the tasks. Pytorch is another Python based library with main emphasis on applications in natural language processing, and its GPU compatibility makes it extremely fast in computationally resourceful servers. Containers consisting of either Tensorflow, Pytorch, or Keras reduces service distribution delay by computing the tasks close to the devices.

In environments such as home automation and autonomous personal assistants, security vulnerabilities lead to cyber attacks like identity theft which can even cause life threatening accidents. To this end, swift deployment and computation of security related services on IoT gateways are of paramount importance while satisfying the latency conditions of the services. Open source containers of IDS services like Snort [41] and Firewall [42] are used in this study to evaluate their performances in IoT gateways. Snort is an intrusion detection and prevention system that identifies vast range of attacks on networks with high accuracy, whereas Firewall provides security by blocking or permitting network traffics to and from the IoT devices. Containerized IDS and Firewall services in IoT gateways enable detection of malicious traffics and proactive blockages of compromised IoT devices. Due to their high computational requirements and significance in IoT systems, this paper uses data analytics and IDS services as representative IoT services, and evaluates their performances in IoT gateways.

### 3.2. Testbed Architecture

**Figure 2:**
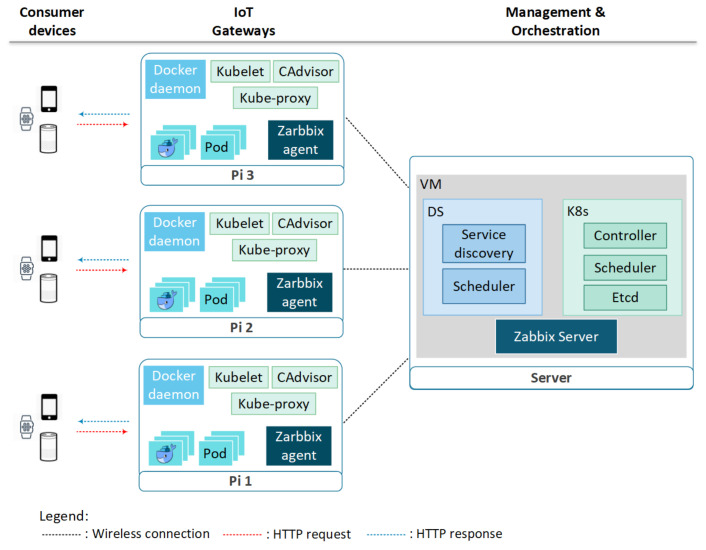
Testbed architecture for docker swarm and kubernetes.

A baseline testbed consisting of a server and three Raspberry Pi boards is developed for this study. The Pi boards operate as IoT gateways in the testbed and wirelessly connect to the server which hosts a DS manager or a K8s Master depending on experiments, as shown in Figure 2. Consumer devices like mobile phones, wearable devices, and virtual assistants act as IoT devices and wirelessly connect to the Pi boards. The orchestrator (i.e., DS manager or K8s Master) is responsible for the dynamic deployment of data analytics and IDS service containers, and wirelessly communicates with the Pi boards. Zabbix, an open source monitoring tool based on client server model, is used for monitoring and gathering relevant container information that includes the creation times of several simultaneous containers, CPU utilization of concurrent containers, and memory usage of containers under different network traffic loads in IoT gateways. The Zabbix server resides in the same server as the orchestrator, whereas Zabbix agents operates in the Pi boards to gather realtime data of different performance metrics and reporting them to the Zabbix server.

The server configuration in the testbed is Intel i7 6700 processor clocked at 3.40 GHz, with DDR4 RAM of size 24 GB, and Ubuntu 16.04 OS. Each IoT gateway is made of a Raspberry Pi3 board with hardware and software configurations as: ARM CPU, internal memory of 1 GB, 2 GB swap memory, Samsung EVO Plus Class 128 GB SD card, and Raspbian Jesse OS. DS version 18.06.1 and Zabbix version 4.0.6 are used in the experiments.

### 3.3. Testbed Configuration

TcpReplay [43] is an open source network traffic regenerating tool using log files of earlier captured traffic. This work utilizes the tool to generate network traffic for the experiments based on bigFlows.pcap [44] file consisting of traffic from real networks, and deploys it inside the Pi boards. The Purpose of using bigFlows.pcap is to emulate the real network traffic at IoT gateways, such that representing real environment in IoT devices. The traffic produced by TcpRelay is directed towards containerized data analytics and IDS services for performance evaluations.

**Figure 3:**
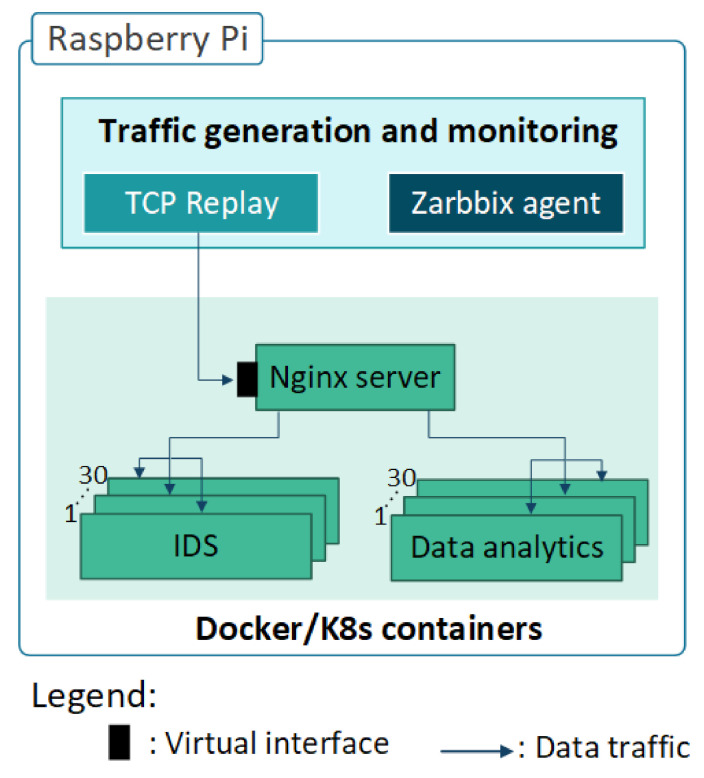
Components of a Pi board for data analytics and Intrusion Detection System (IDS) services experiments with docker swarm and kubernetes.

Components of a service container in a Pi board are depicted in Figure 3. Network traffic from TcpReplay is fed into Nginx server container first. Nginx container replicates the same traffic and send it to all available containers in the Pi board. Depending on each experiment, individual or various instance(s) of a service container are deployed in an IoT gateway at a given time. Snort and Firewall containers are representatives of IDS services, where data analytics services are presented by Pytorch, Tensorflow, and Keras containers in our experiments. Network traffic is processed by IDS and data analytics service containers depending on the experiment while Zabbix agent gathers performance data. Containers of each service are increased in each experiment, till 30, and for each iteration of the experiment, performance metrics are monitored and logged.

## 4. Performance Evaluation

### 4.1. Methodology

A single server and three Pi boards were used in all the experiments. Performance metrics for DS and K8s were measured against increasing service container instances. In each experiment, there could be several service container instances operational in three Pi boards, and their deployment distribution is shown in Table 1. Performance metrics included container creation time, CPU utilization, memory usage, and energy consumption. Creation time of a container was the summation of time required for retrieving an image file from the container image repository, and deploying the image on a Pi board, and reaching ready state of the container. In the experiments, service containers were deployed based on the aforementioned sequence in Table 1 and the delay of such concurrent deployments was monitored. Operators could utilize the results of time taken by particular service container to be in ready state, for appropriately adjusting the auto scaling threshold to avoid service degradation from container overloading.

In a single experiment, network traffic was processed by the container of a particular service and average CPU utilization of each Pi board was measured. Service containers were incremented in each experiment using the same criteria as creation time experiment, to examine the effects of the increment on CPU utilization of resource constrained device. Similar experiments were used to measure the energy consumption of the service containers in Pi boards. Information regarding number of containers and the traffic load that an IoT gateway could support under QoS and energy consumption constraints, enabled proper network planning for service provisioning. Additionally, for maintaining QoS of a service, it was important that containers of every service received a fair amount of memory, and it was investigated through monitoring the memory usage of different service containers while processing the network traffic. Memory usage of each service container was measured against network traffic of 10 Mbps, 50 Mbps, 70 Mbps and 100 Mbps. Every result shown in the graphs is an average value of five experiments, and each CPU utilization and memory usage experiment were carried out for 10 h. Analysis of creation time, CPU utilization, and memory usage results for both IDS and data analytics containerized services with DS and K8s are discussed in subsequent subsections.

### 4.2. Container Creation Time for IDS and Data Analytics Services

**Figure 4:**
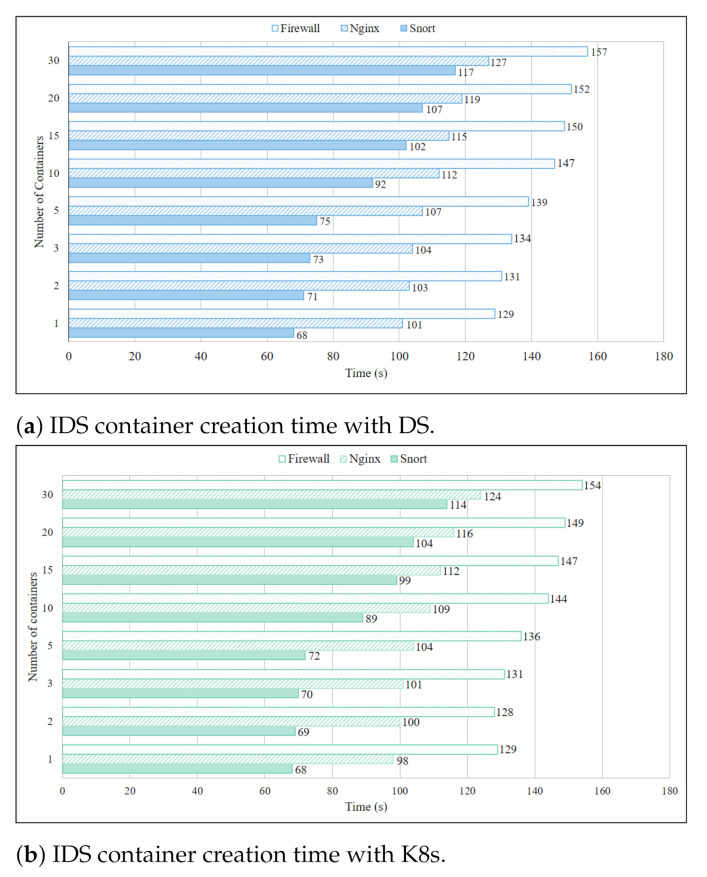
IDS container creation time with DS.

Results for IDS service container creation time on three Pi boards with DS are depicted in Figure 4a. Firewall service containers with relatively large image files took the most time for creation, because pulling large files from the repository took much time. To create 30 service containers, Firewall service took 157 s, Snort service takes 117 s, and Nginx service took 127 s. This concludes that in general an IDS service took approximately 4 min to be created on three IoT gateways. Another observation was that the increase in the creation time of all the IDS service was proportional to the increase in container instances, and it held if the container instances further increased. Results in Figure 4b depict the K8s performance for container creation time of the same services and experiment settings. Since an image file was used, like DS, it can be seen that K8s was about 2–3 s faster than DS. The creation time of 30 service containers of Firewall was 154 s, Snort was 114 s, and Nginx was 124 s, exhibiting that K8s took approximately three minutes to create a general IDS service on three IoT gateways. Similarly to DS, creation time of K8s based IDS services increased proportionally to the instances of containers, and this trend continued if the number of instances was further increased.

**Figure 5:**
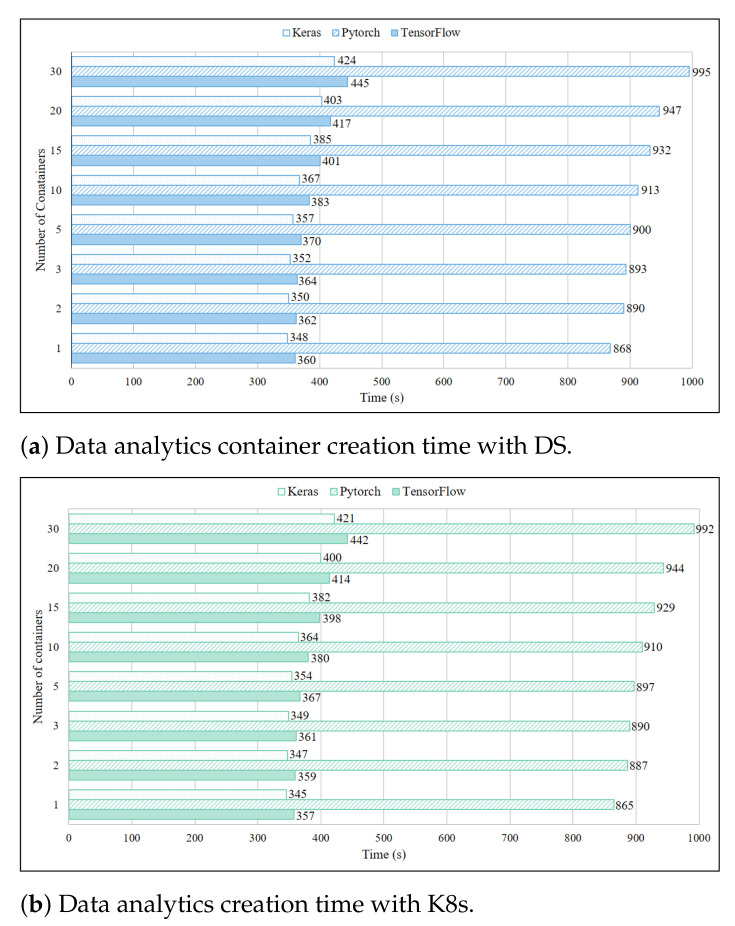
Data analytics container creation time with DS.

Data analytics services experiments were done with TensorFlow, Pytorch and Keras, and Figure 5a shows their creation time when deployed in three Pi boards with DS. In comparison to IDS service results, data analytics services took a lot more time for creation because of the larger image files. Pytorch was an exception among these services as it had extraordinarily long creation time due to the same reason. However, similar patterns could be traced in the IDS services and data analytic services creation time. The same image files and experiment settings were used to measure the K8s performance in Figure 5b. As expected K8s showed slightly better performance than DS which was consistent with the performance in case of IDS service containers. It can be deduced from the results that increase in the image file size linearly increased the creation time. This experiment results indicated the crucial role that creation time plays in timely scaling of service container.

### 4.3. CPU Utilization for IDS and Data Analytics Services

**Figure 6:**
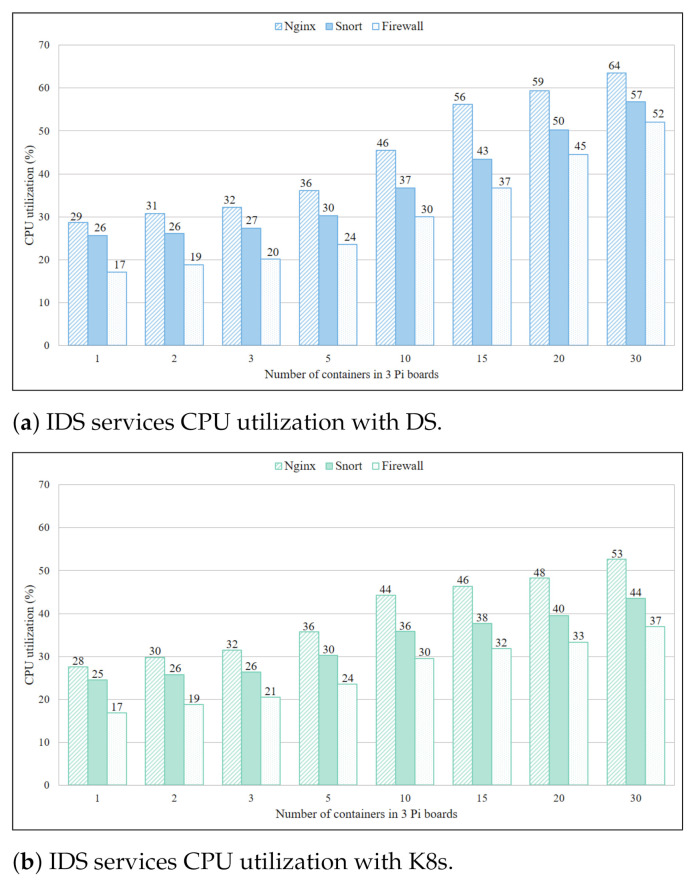
IDS services CPU utilization with DS.

Mean CPU utilization of IDS services for three Pi boards while using DS is shown in Figure 6a, where utilization consistently increases with increasing IDS service container instances. Mean CPU utilization of 30 service containers, deployed across three Pi boards, is 63.6% for Nginx, 56.8% for Snort, and 52.1% for Firewall. It is recorded that in one Pi board CPU utilization increments 15.3% with addition of five more containers. If this pattern is extrapolated, a Pi board becomes overloaded with 67.3% utilization when five more container instances are added. Other IDS services follow the similar pattern, and from this it can be inferred that instances of IDS service containers can go up to 10 in IoT gateway for the usual utilization threshold of 65%. Mean CPU utilization results for IDS services with K8s on three Pi boards, in Figure 6b, show a similar pattern but improved performance. The mean CPU utilization for Nginx is 52.63%, for Snort it is 43.51% and for Firewall it is 37.02%. Comparing it against the DS Nginx uses 10.94% less, Snort uses 13.26% less, and firewall uses 15.09% less. Based on these K8s results, it can be inferred that containers of more than 20 services can operate on each board without overload.

**Figure 7:**
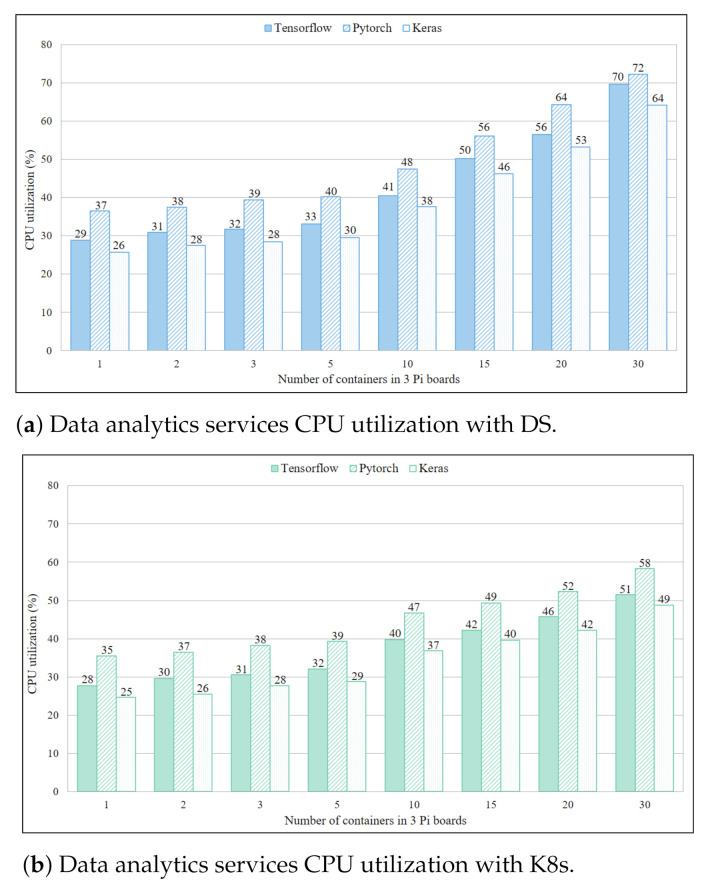
Data analytics services CPU utilization with DS.

As presented in Figure 7a, CPU utilization of DS based data analytic service containers was only slightly higher in comparison to IDS services despite the high creation time due to the size of image files. The general notion of data analytic services being computationally too expensive for resource constrained devices was negated by these results. The CPU utilization values in Figure 7b for TensorFlow, Pytorch and Keras with 10 containers in a single Pi board (overall 30) were 69.7%, 72.3% and 64.3%, respectively, and that was over the utilization threshold of 65%. This indicated that to avoid overloading in IoT gateway only five to seven data analytic service containers should operate simultaneously. Similar to the case of IDS services, K8s showed better CPU utilization than DS in Figure 7b. The CPU utilization values for TensorFlow, Pytorch and Keras with 10 containers in a Pi board are 51.48%, 58.29% and 48.79%, respectively. Based on these results, it can be inferred that by using K8s containers more than 10 services could operate on each board without overloading.

### 4.4. Memory Usage for IDS and Data Analytics Services

**Figure 8:**
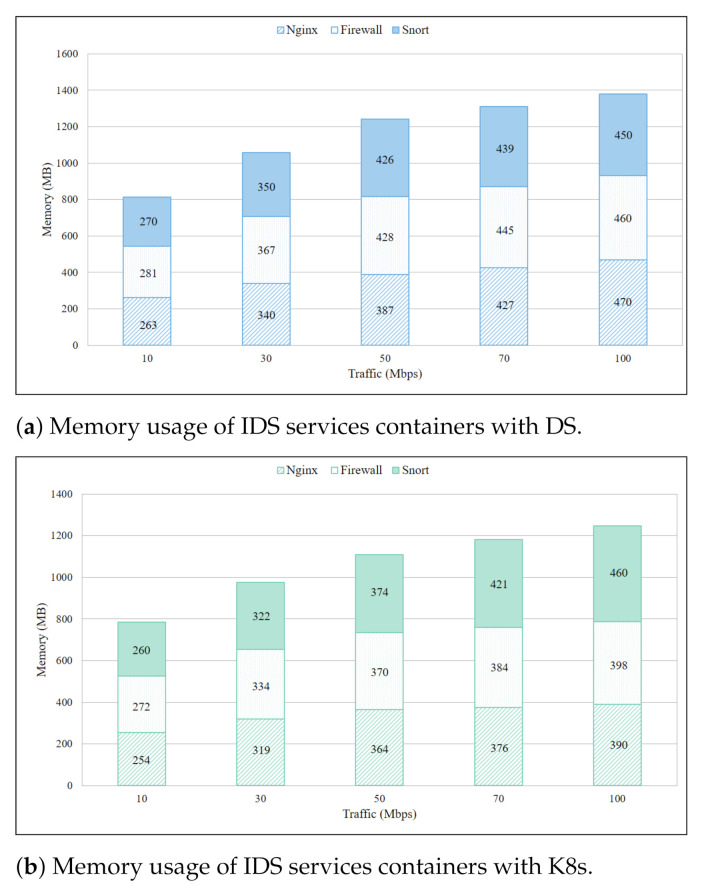
Memory usage of IDS services containers with DS.

Memory usage of a single DS container per IDS service in a single Pi board is presented in Figure 8a where all the containers were deployed simultaneously. Equal network traffic was routed to these containers for concurrent computing. The rationale of this experiment was to examine the memory distribution among containers of different services, and the results demonstrate that memory consumption was almost similar for all IDS service containers. Moreover, steady increment in per service memory usage was observed with the increment in traffic. The total memory consumption approached 1.380 MB with 100 Mbps which was 46% of total memory. The same experiment with K8s revealed total memory usage of 1.248 MB in Figure 8b when 100 Mbps traffic was directed towards individual containers. Which is 132 MB less in comparison to DS. Other results confirmed that on average K8s used 101 MB less memory than DS. Insight from this experiment was that memory in the IoT gateway was rapidly exhausted when deployed service containers got heavy network traffic. To avoid memory starvation and keep IoT gateway operational, one of the ways was to move service containers with heavy traffic load to Fog or cloud servers.

**Figure 9:**
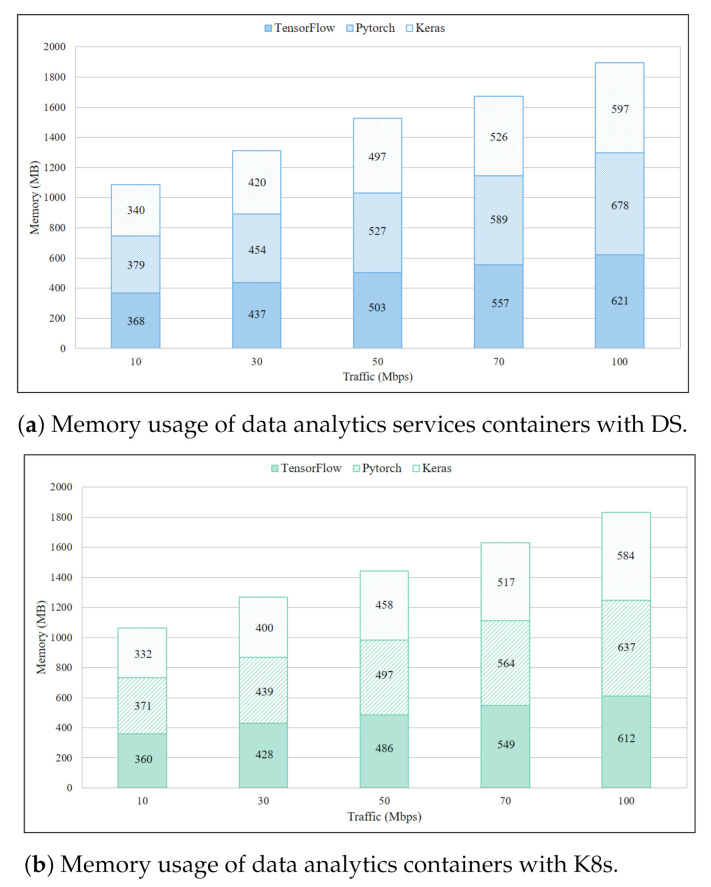
Memory usage of data analytics services containers with DS.

Memory consumption of data analytic services is higher than IDS services for both DS and K8s, as shown in Figure 9a,b, respectively. Results of both DS and K8s also revealed an even distribution of memory usage among different containerized services, irrespective of the service types. K8s showed on average 51.8 MB less memory consumption than DS, which could be crucial in extremely resource constrained IoT systems. A more crucial observation from these experiments was that for merely 100 Mbps traffic the memory consumption by each service container was quite substantial. With multiple active service containers under high traffic this could cause disruption in IoT gateway operations and severely degrade QoS. To avoid this from happening a comprehensive monitoring system was required which triggered auto scaling of services once the defined threshold was reached.

### 4.5. Energy Consumption for IDS and Data Analytics Services

Energy consumed by DS and K8s for IDS and data analytics services was calculated through a power consumption model [23] that uses CPU utilization, and it is defined as:

(1) $$P_{CPU}=1.5778W+0.181\mu W,$$

where μ is the CPU utilization between 0 and 1. Power consumed by a Pi board in computing a service container over time is defined as energy consumption, and it is expressed as:

(2) $$E_{kJ}=3.6tP_{CPU}.$$

Cumulative energy consumption of three Pi boards is computed using (1) and (2) for different DS and K8 service containers deployments as per Table 1. As each experiment is conducted for 10 h, the *t* is set to 10 in (2).

**Figure 10:**
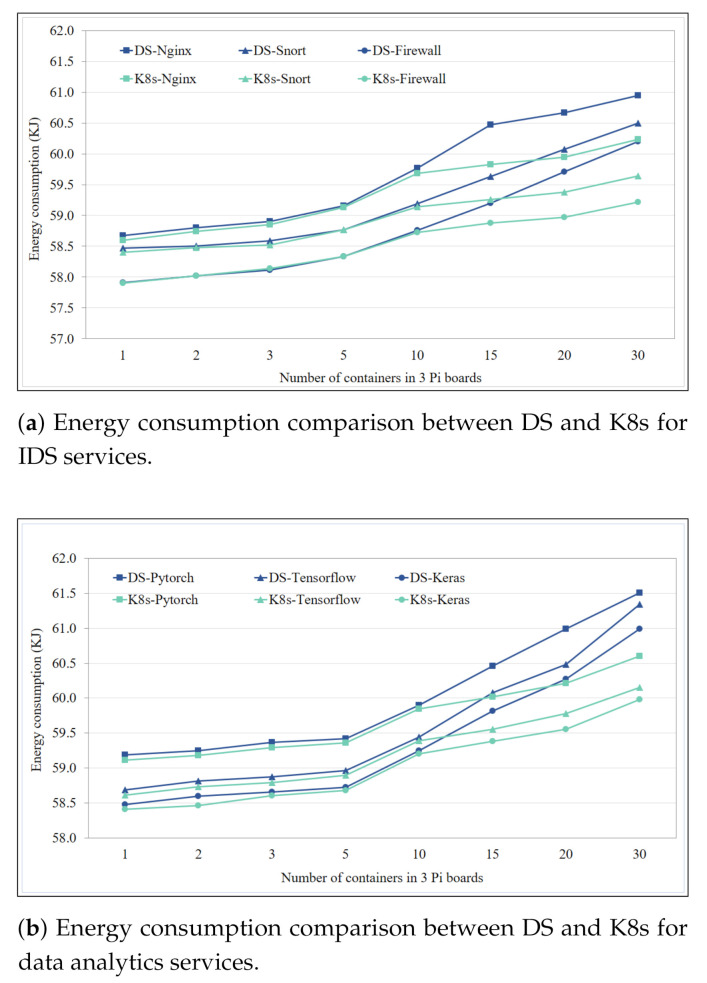
Energy consumption comparison between DS and K8s for IDS services.

Energy consumption comparison between DS and K8s for IDS services containers is shown in the Figure 10a. Increment pattern of energy consumption for all three IDS services, Nginx, Snort, and Firewall was almost similar when deployed with DS, where Nginx consumed the most energy 61 kJ with 10 containers in a single Pi board. Energy consumption of same services with K8s was similar to DS till there are three containers in a single Pi board (overall 10), from this point increment in energy consumption with addition of more containers was reduced. With 10 containers in a Pi board (overall 30), Nginx, Snort, and Firewall service containers with K8 showed 1.15%, 1.49%, and 1.67% less energy consumption than DS, respectively. For reference a smart phone in an idle state consumes 0.9 kJ energy in 10 h [45]. This makes K8s much more energy efficient than DS for IDS services.

Energy consumed by DS and K8s for data analytics services containers is shown in the Figure 10b. Both DS and K8s showed sharp increase in energy consumption for all data analytics services when the number for containers per Pi board became more than two. Post sharp increase, DS based data analytics service containers maintained this increment almost linearly as more number of containers were added. However, similar to the case of IDS services, increment in energy consumption of K8s data analytics service containers was reduced once the number of containers per Pi board become more than three. Pytorch, Tensorflow, and Keras service containers based on K8s consumed 1.63%, 1.97%, and 1.65% less energy than DS service containers, respectively, when 10 containers were deployed per Pi board (overall 30). This translated into 0.91 kJ, 1.19 kJ, and 1.01 kJ less energy consumed by K8s Pytorch, Tensorflow, and Keras service containers over 10 h, and this could significantly prolong the life time of a battery operated IoT device or gateway.

## 5. Concluding Discussion and Future Work

This paper presents performance analysis of different containers on resource constrained devices and compares the results of two orchestration systems namely DS and K8s. IDS and data analytics services are used as two use cases and multiple open source solutions of these services are evaluated in the experiments. A baseline testbed with three Raspberry Pi boards serves for the experiments and results show that K8s outperforms DS in all three aspects of container life cycle management that are analyzed in this paper. Specifically, K8s shows average 3 s less container creation time, 14.5% less CPU utilization, and 76.4 MB less memory usage. Results from this paper contribute towards fine tuning of operators’ service scaling and provisioning policies for low latency and high availability use cases like virtual assistants, video surveillance, V2X offloading, and industry automation. Specifically, the container creation time results reveal a direct relationship with container image file and consistently increase as the number of containers go up. In terms of CPU utilization, more than 30 IDS service containers and 13 data analytics service containers can be accommodated with K8s. Moreover, memory consumption results show service agnostic characteristic for memory distribution among different containers in a single Pi board, but worryingly the number of service containers that can operate in an IoT gateway under moderate traffic load are three to five. This problem can be solved by defining thresholds in advance and performing auto scaling of services according to policies. In terms of energy consumption, difference between DS and K8s is minuscule till three containers are deployed per device. For deployment of more than three service containers per device, K8s should be used to conserve the energy. In summary, network operators should consider the average container creation time of 311 s while deploying and scaling a service. At a given time maximum of five service containers should be deployed in resource constrained devices under moderate traffic load of 100 Mbps due to memory limitation. All the experimental results indicate that K8s is a more robust and efficient solution for implementing dynamic services in IoT gateways. Our future studies will evaluate more real word situations where functions like traffic anomaly detection, speech synthesis, and load prediction are dynamically deployed using different containers orchestration solutions.

## Figures and Tables

**Table 1:** Deployment distribution of containers in three Raspberry Pi boards.

Total No. of Containers	In Pi 1	In Pi 2	In Pi 3
1	1	0	0
2	1	1	0
3	1	1	1
5	2	2	1
10	3	3	4
15	5	5	5
20	7	7	6
30	10	10	10

## Data Availability

All the data generated during the experiments is presented in the article.
